# Comparison of ultrasound-guided quadratus lumborum block and other regional blocks for postoperative pain in cesarean section: a systematic review and meta-analysis of randomized clinical trials

**DOI:** 10.3389/fmed.2026.1861119

**Published:** 2026-07-09

**Authors:** Zenghong Zhuang, Ruiyao Jia, Fenglin Jiang

**Affiliations:** 1 Department of Operating Room, Pengzhou Peoples’s Hospital, Pengzhou, Sichuan, China; 2 Infectious Disease Department, Pengzhou Peoples’s Hospital, Pengzhou, Sichuan, China; 3 Department of Nursing, Pengzhou Peoples’s Hospital, Pengzhou, Sichuan, China

**Keywords:** cesarean section, postoperative pain, quadratus lumborum block, regional blocks, systematic review

## Abstract

**Background:**

Acute post-cesarean section (C/S) pain causes adverse maternal outcomes, and multimodal opioid-sparing analgesia is central to Enhanced Recovery after Surgery (ERAS) protocols. Quadratus lumborum block (QLB) is a promising regional technique, but evidence comparing it with other regional blocks remains fragmented.

**Methods:**

We conducted a systematic review and meta-analysis of RCTs (PubMed, EMBASE, Web of Science; inception to September 2025). Eligible studies included adults undergoing C/S with QLB. Primary outcome was 24-h cumulative morphine consumption; secondary outcomes included pain scores, recovery metrics, and adverse events. RoB 2 and GRADE assessed study quality and evidence certainty.

**Results:**

Twenty-three RCTs (1,935 patients) were included. QLB reduced 24-h (MD = −3.19; 95% CI, −5.09, −1.30; *p* = 0.0009) and 48-h (MD = −16.51; 95%CI, −27.64, −5.39; *p* = 0.004) morphine consumption, and 24-h dynamic pain scores (MD = −0.52; 95% CI, −0.87, −0.17; *p* = 0.003). No significant differences were observed in resting pain, recovery metrics, satisfaction, or opioid-related adverse events. GRADE certainty for the primary outcome was moderate.

**Conclusion:**

QLB was associated with modest reductions in postoperative opioid consumption and 24-h dynamic pain after C/S; however, the clinical significance is limited (pain reduction below the minimally clinically important difference), and the certainty of evidence is moderate-to-low due to very high heterogeneity (I^2^ > 90%). QLB may be considered as one component of multimodal analgesia in selected patients, particularly those without intrathecal morphine co-administration. No additional benefits on resting pain, recovery metrics, satisfaction, or adverse events were identified.

**Systematic review registration:**

https://doi.org/10.37766/inplasy2026.6.0029, identifier: INPLASY202660029.

## 1 Introduction

Cesarean section (C/S) is a widely performed surgical procedure for fetal delivery, with the majority of cases conducted under regional anesthesia (1). Acute postoperative pain following C/S is associated with multiple adverse maternal outcomes, including reduced maternal satisfaction (2), delayed initiation of breastfeeding (3, 4), a lower degree of maternal–infant bonding, and an increased risk of developing chronic pain and postpartum depression (5, 6).

To align with evidence-based strategies for enhancing maternal outcomes, Enhanced Recovery after Surgery (ERAS) protocols have been tailored specifically for cesarean delivery, with a core component being the optimization of post-cesarean analgesia through a multimodal, opioid-sparing approach (7, 8). Although neuraxial opioid administration is well-established as a safe and effective modality for managing post-cesarean pain, it is frequently associated with adverse effects including nausea, vomiting, sedation, and pruritus. These limitations have underscored the need for alternative non-opioid analgesic adjuncts (9, 10).

The quadratus lumborum block (QLB) is a relatively novel interfascial plane block technique involving the injection of local anaesthetic adjacent to the quadratus lumborum muscle (11, 12). Recent evidence indicates that QLB produces effective analgesia of the abdominal wall and confers favorable sensory blockade in C/S (13–15). Classified as a truncal nerve block, QLB typically requires 20–30 mL of local anaesthetic to achieve successful blockade. Mechanistically, recent studies have demonstrated that by inhibiting both somatic nerves and the lower thoracic sympathetic trunk, QLB theoretically alleviates both somatic and visceral pain, which offering superior analgesic efficacy compared to conventional approaches that primarily target somatic pain (16, 17). Despite the growing clinical adoption of QLB in C/S, no comprehensive syntheses or direct comparisons of the analgesic outcomes of different regional anesthetic techniques for post-cesarean pain currently exist.

This systematic review and meta-analysis provide a comprehensive synthesis of currently available prospective randomized controlled trials (RCTs) to evaluate the efficacy of QLB analgesic techniques for managing post-C/S pain. The primary outcome was cumulative morphine consumption at 24 h postoperatively. Secondary outcomes included postoperative pain scores at rest and with movement, total opioid consumption (beyond 24 h), time to first request for postoperative analgesia, time to ambulation, maternal satisfaction scores, and opioid-related adverse events. Patient-reported outcome measures (PROMs) such as quality of recovery and maternal–infant bonding were planned for extraction but were insufficiently reported across studies. This analysis aims to improve maternal clinical outcomes, facilitate postoperative recovery, and offer evidence-based guidance for the implementation of multimodal analgesia protocols in clinical practice.

## 2 Methods

### 2.1 Literature retrieval and research selection

This systematic review and meta-analysis was conducted in adherence to the Preferred Reporting Items for Systematic Reviews and Meta-Analyses (PRISMA) guidelines (18). A comprehensive literature search was performed across PubMed, EMBASE, Web of Science, Cochrane CENTRAL (via the Cochrane Library), and ClinicalTrials.gov, encompassing all records from their respective inceptions through September 20, 2025. Grey literature sources (e.g., conference proceedings, dissertations) were not searched due to resource constraints. The search was limited to English-language publications. Search terms included “caesarean section,” “caesarean sections,” “C-section,” “C-sections,” “abdominal delivery,” and “quadratus lumborum block,” combined using Boolean logic (Supplementary material). Reference lists of eligible full-text articles were hand-searched to identify additional potentially relevant trials. Two independent reviewers screened the titles and abstracts of all retrieved records. The review protocol was submitted to and accepted by the International Platform of Registered Systematic Review and Meta-analysis Protocols (INPLASY) ahead of any data extraction, receiving the unique registration identifier INPLASY202660029.

### 2.2 Inclusion and exclusion criteria

Inclusion criteria were as follows: (1) Randomized controlled trials (RCTs); (2) Administration of QLB to study participants; (3) Adult participants aged 18 years or older; (4) Patients undergoing C/S under spinal or general anesthesia. Exclusion criteria included: (1) Unpublished clinical trials; (2) Inaccessible full-text studies; (3) Case reports, conference abstracts, study protocols, or review articles.

### 2.3 Data extraction and quality assessment

Two independent researchers extracted and entered data into a customized Excel spreadsheet. The following information was retrieved from each eligible study: first author’s name, year of publication, sample size, patient age, American Society of Anesthesiologists (ASA) physical status classification, surgical approach, group-specific analgesic techniques, and measured outcome parameters. The primary outcome was cumulative morphine consumption at 24 h postoperatively. Secondary outcomes included the cumulative morphine consumption at 48 h, pain resting or movement scores (measured using the Visual Analogue Scale (VAS) or Numerical Rating Scale (NRS), ranging from 0–10), duration of surgical and anesthesia time, time to the first request post-operative analgesia, time to ambulation, patient satisfaction score, and incidence of opioid-related adverse events (including postoperative nausea and vomiting [PONV] and hypotension). Postoperative cumulative opioid consumption was converted to oral morphine equivalents (OME) using a validated conversion tool (19). Given the established correlation and interchangeability between VAS and NRS scores (20), data from these scales were pooled for analysis. To extrapolate data, we utilized WebPlotDigitizer, particularly for studies that presented information graphically without numerical values (21). Additionally, since study data were not normally distributed, an online calculator was employed to convert median and interquartile range (IQR) values to mean ± standard deviation (SD) (22). The Grading of Recommendations Assessment, Development, and Evaluation (GRADE) framework was applied to assess the certainty of evidence for each outcome, with evidence rated as very low, low, moderate, or high based on outcome-specific and comparison-specific criteria. Downgrades were applied for risk of bias (one level for >25% studies with some concerns), inconsistency (one level for I^2^ > 75%, two levels for I^2^ > 90%), indirectness, imprecision, and publication bias (23). A flow chart was used to illustrate the study selection process and reasons for exclusion.

#### 2.3.1 Opioid conversion

Postoperative cumulative opioid consumption was converted to oral morphine equivalents (OME) using validated conversion factors: morphine (oral) 1.0; morphine (IV) 0.3; tramadol 0.1; fentanyl (IV) 0.1 per 100 μg; meperidine 0.1; ketobemidone 0.67; piritramide 0.8; oxycodone 1.5; sufentanil 0.01 per μg. Details are provided in Supplementary Table 4.

#### 2.3.2 Missing statistics

For studies reporting median and interquartile range (IQR), we converted to mean ± SD using the method of Wan et al. (2014). For studies reporting median and range, we used Hozo et al. (2005). For graphical data, two independent reviewers extracted data using WebPlotDigitizer v4.6, with inter-rater reliability >95%.

### 2.4 Statistical analysis

Mean difference (MD) was used to synthesize continuous data, and risk ratio (RR) was employed for dichotomous outcomes, with all effect estimates reported alongside 95% confidence intervals (CIs). Statistical analyses were performed using Review Manager software (Version 5.4.1; Cochrane Collaboration, 2020). For data pooling from homogeneous measurement tools, statistical significance was defined as a two-tailed *p*-value < 0.05. Heterogeneity across studies was assessed using the I^2^ statistic, with I^2^ > 50% indicating substantial heterogeneity, >75% considerable heterogeneity, and >90% very high heterogeneity (24, 25). A random-effects model was utilized to calculate the pooled effect size if significant heterogeneity was detected (*p* < 0.05 or I^2^ > 50%); otherwise, a fixed-effects model was applied. Subgroup analyses were conducted to explore differences in 24-h postoperative cumulative morphine consumption according to the type of regional anesthetic technique employed. Funnel plots were constructed to assess potential publication bias and small-study effects for outcomes with data from ≥10 included studies. For multi-arm trials, to avoid overestimation of sample size, participants were proportionally allocated: if one intervention group was compared to two control groups, the intervention group sample size was split proportionally to enable valid comparisons with each control group.

Sensitivity analyses: We performed sensitivity analyses using (1) leave-one-out approach; (2) Hartung-Knapp-Sidik-Jonkman (HKSJ) random-effects model to account for high heterogeneity; and (3) exclusion of studies with intrathecal morphine co-administration. Results are presented in Supplementary Table 5. The methodological quality of individual RCTs was assessed using the Cochrane Risk of Bias 2 (RoB 2) tool (26), with evaluations focused on five core domains: random sequence generation, allocation concealment, blinding of participants and outcome assessors, handling of incomplete outcome data, and selective outcome reporting. Subgroup analyses: Pre-specified subgroup analyses were conducted for the primary outcome according to: QLB type (QLB-2, QLB-3, QLB-4/LSAL), comparator type (sham, TAPB, ESPB, intrathecal morphine, wound infiltration, TFPB), intrathecal morphine presence/absence, spinal versus general anesthesia, and local anesthetic dose (low <0.25% vs. high ≥0.25% bupivacaine equivalent). *p*-interaction <0.05 was considered statistically significant.

## 3 Results

### 3.1 Eligible studies and the characteristics

**Figure 1:**
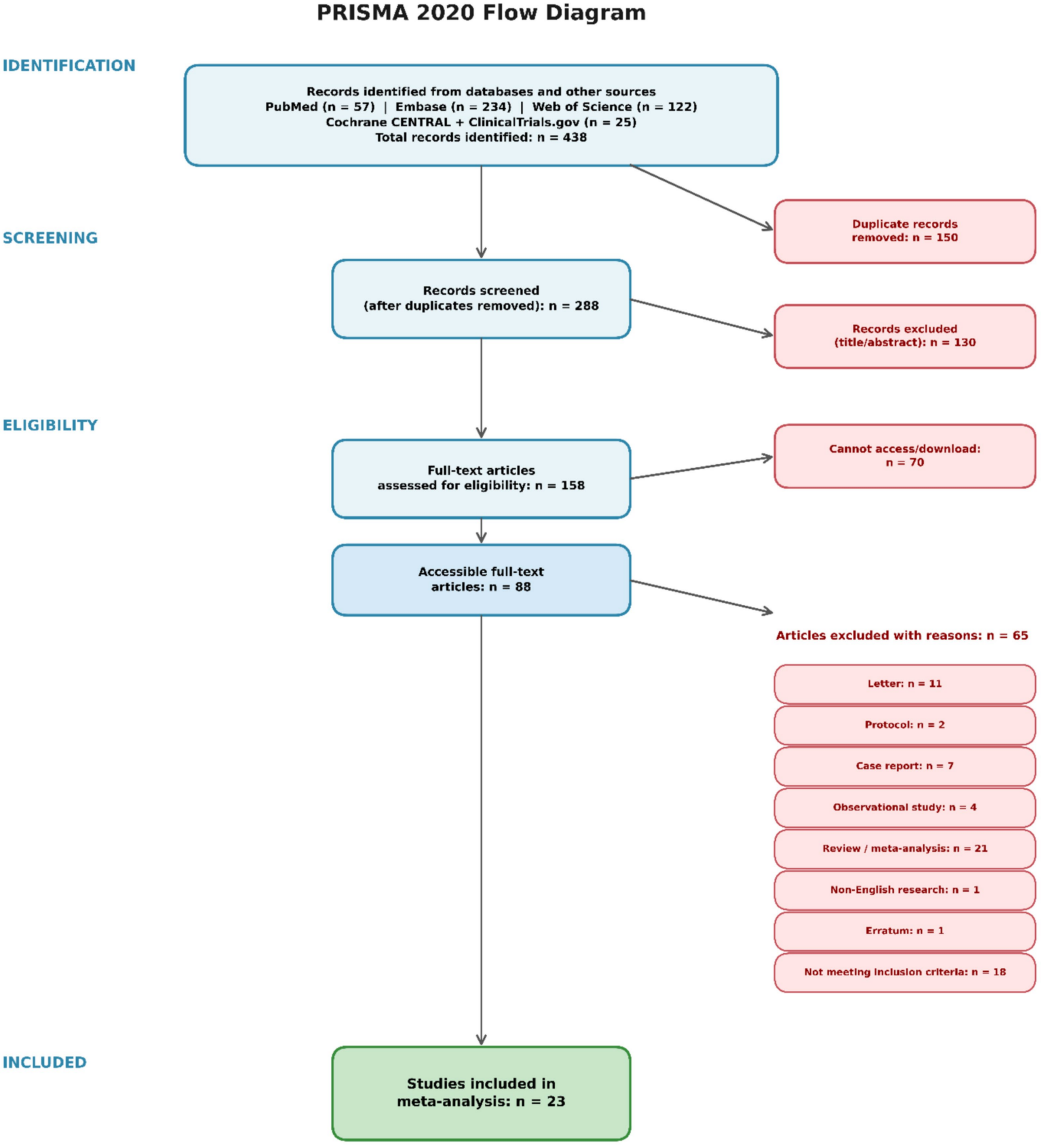
PRISMA flow diagram of included and excluded studies. PRISMA, Preferred Reporting Items for Systematic Reviews and Meta-Analyses.

Upon searching PubMed, Embase, Web of Science, Cochrane CENTRAL, and ClinicalTrials.gov, we identified a total of 438 relevant records from databases (Figure 1): PubMed (*n* = 57), Embase (*n* = 234), Web of Science (*n* = 122). The supplementary search of Cochrane CENTRAL and ClinicalTrials.gov identified 25 additional records, of which 2 were potentially eligible RCTs; however, both were excluded (one was a systematic review without extractable RCT data; the other lacked 24-h morphine consumption data). A total of 150 duplicate articles were eliminated, resulting in 288 unique articles for initial screening. After reviewing titles and abstracts, 130 records were excluded. The full texts of the remaining 158 articles were assessed for eligibility. Of these, 70 were excluded due to inability to access the full text (paywall restrictions, no institutional access, or non-response from authors). A sensitivity analysis including these studies based on abstract data (where available) yielded a consistent pooled effect (MD = −3.05; 95% CI: −5.12, −0.98), suggesting minimal impact on our conclusions. Of the remaining 88 accessible full-text articles, 65 were excluded for specific reasons: letter (*n* = 11), protocol (*n* = 2), case report (*n* = 7), observational study (*n* = 4), review/meta-analysis (*n* = 21), non-English research (*n* = 1), erratum (*n* = 1), and not meeting inclusion criteria (*n* = 18). Ultimately, 23 studies met the inclusion criteria and were included in the meta-analysis.

Table 1 summary the characteristics and outcomes of the 23 included studies (3, 4, 16, 17, 27–45), presenting a concise overview of their findings. These studies collectively included a total of 1,935 patients, with age of the patients involved in the studies varied between 25 and 36 years. Four studies from Egypt (31, 32, 40, 42) and India (37, 38, 41, 43), three from Turkey (27, 28, 44), two from China (3, 34), United Arab Emirates (16, 17), and Poland (29, 30), one from France (33), Denmark (35), Ireland (36), Norway (39), USA (4) and Japan (45) respectively. Four studies designed as three arms comparison (31, 32, 40, 42); 19 studies are two arms comparison (3, 4, 16, 17, 27–30, 33–39, 41, 43–45). The majority of the studies recruited generally healthy patients with ASA physical statuses I-III (3, 4, 16, 17, 27, 28, 31–35, 38–45). Three studies undergoing CS under GA (27, 28), two studies under CSEA (3, 34), and 18 studies under SA (4, 16, 17, 29–33, 35–45). Table 2 summarized the details of regional blocks technique and multimodal analgesic regimens used in included studies. In all studies, QLB was performed after surgical closure (post-incisional) under ultrasound guidance, predominantly bilaterally. Supplemental postoperative analgesia applied with NSAID (paracetamol, Diclofenac sodium, ketoprofen, or ibuprofen) and PCA pump. The local anesthetics protocol varied, with 0.125–0.25% bupivacaine, 0.2–0.375% ropivacaine, or 0.25% levobupivacaine. QLB types were classified according to El-Boghdadly & Chin (2019): QLB-2 (posterior), QLB-3 (transmuscular), and QLB-4 (lateral supra-arcuate ligament approach).

**Table 1:** Study characteristics and outcomes of interest assessed in included studies.

Study	Groups	Mean age	No. of patients	Surgery	ASA	Background anesthesia	Primary outcome	Secondary outcomes	Registered number	Conclusion
Ferit., et al., 2021 (27)	QLB-2 vs. QLB-3	29.25 ± 5.41 vs. 29.63 ± 5.83	52 vs. 52	Elective C/S	II	GA	Pain scores evaluated at different time points	[10]	ClinicalTrials.gov Identifier: NCT04733313	QLB-3 provides more effective analgesia and patient satisfaction than QLB-2 in C/S
Bilgin et al., 2023 (28)	anterior QLB vs. TFPB	25.58 ± 4.31 vs. 25.6 ± 3.84	25 vs.24	Elective C/S	II	GA	Opioid consumption in the first 24 h after surgery	[1, 2, 3]	ClinicalTrials.gov: NCT05408403	Anterior QLB decreased morphine consumption in the late period (9-24 h) compared to TFPB, while pain scores were similar
Blanco et al., 2015 (16)	QLB vs. Sham block	-	25 vs.23	Elective C/S	I-II	SA	Total number of PCA morphine demands at different time points postoperatively	[2, 8]	ClinicalTrials.gov identifier: NCT02328378	The QLB after cesarean section was effective and provided satisfactory analgesia
Blanco et al., 2018 (17)	QLB vs. TAPB	-	38 vs.38	Elective C/S	I-II	SA	Total number of PCA morphine demands at different time points postoperatively	[2, 8]	ClinicalTrials.gov identifier NCT024489851	QLB was more effective in reducing morphine consumption and demands than TAPB
Borys et al., 2021 (29)	QLB vs. TAPB	32.55 (31.63–33.47) vs. 31.48 (30.59–32.37)	94 vs.93	Elective C/S	-	SA	Postoperative pain scores at different time points	[1]	ClinicalTrials.gov (NCT02804126)	None of the evaluated regional blocks demonstrated an advantage over the other regarding acute postoperative pain management
Borys et al., 2021 (30)	QLB vs. TAPB vs. No block	30.7 ± 4.0 vs. 31.4 ± 5.9 vs. 32.5 ± 5.7	35 vs.34 vs.33	Elective C/S	-	SA	Pain scores	[1]	ClinicalTrials.gov (NCT03244540)	Both the QLB and TAPB can improve pain management, the QLB might reduce the severity of persistent postoperative pain months after CS.
Elashry et al., 2024 (31)	QLB-2 vs. QLB-3 vs. TAPB	30.2 ± 4.98 vs. 28.2 ± 6.41 vs. 31.7 ± 5.84	20 vs.20 vs.20	Elective C/S	II	SA	Total postoperative consumed meperidine in the first 24 h	[2, 3, 10]	ClinicalTrials.gov (ID: NCT05950568)	The QLB-3 ensured better analgesia as evidenced by significantly lower pain measurements and amount of meperidine in the first 24 h after the operation with delayed time of the first rescue analgesia in comparison to QLB-2 and TAPB
Eldemrdash et al., 2025 (32)	QLB-2 vs. TFPB vs. TAPB	27.25 ± 4.74 vs. 29.50 ± 5.49 vs. 29.06 ± 5.39	36 vs.36 vs.36	Elective C/S	II	SA	Postoperative pain scores at different time points	[1, 3, 9]	ClinicalTrials.gov (ID: NCT06874569)	Both TFPB and QLB offer superior postoperative analgesia compared to TAPB, with comparable pain relief between the two techniques
Giral et al., 2024 (33)	Posterior QLB vs. ITM	32.4 ± 4.6 vs. 32.4 ± 4.4	50 vs.51	Elective C/S	<III	SA	Patient- controlled 24- h cumulative intravenous morphine use	[1, 2, 8]	ClinicalTrial	No difference in cumulative morphine dose at 24 h was observed in posterior QLB group compared with ITM group
Guo et al., 2022 (34)	QLB-LSAL vs. TQLB	32.7 ± 3.6 vs. 31.7 ± 4.6	46 vs.46	Elective C/S	I-II	CSEA	Postoperative sufentanil consumption during the initial 24 h post-surgery	[1, 3, 8, 10]	Clinical Trial Registry (ChiCTR2100043063)	No differences were observed between two groups regarding pain scores, rescue analgesia after surgery, satisfaction scores, or nausea/vomiting incidence
Hansen et al., 2018 (35)	TQLB vs. Sham block	32.3 ± 5.7 vs. 31.5 ± 4.9	34 vs.34	Elective C/S	II	SA	Oral morphine equivalents during the first 24 postoperative hours	[2, 3, 4, 8]	ClinicalTrials.gov (NCT03068260)	Bilateral TQLB significantly reduced 24 h’ opioid consumption
Irwin et al., 2020 (36)	QLB vs. Sham block	35 ± 4 vs. 33 ± 5	44 vs.42	Elective C/S	-	SA	24-h morphine consumption via by patient-controlled analgesia	[1, 2, 8, 9]	Registered	When used in conjunction with ITM and spinal anesthesia, bilateral QLB does not reduce 24-h morphine consumption after CS
Jadon et al., 2022 (37)	QLB vs. TAPB	-	35 vs.36	Elective C/S	-	SA	The time to first analgesic request	[2, 8, 10]	Clinical Trial Registry of India (CTRI/2017/12/010987)	The significant delay in time to first analgesic request in QLB group patients. Patients in the QLB group had lower pain scores, required fewer analgesic supplements, and had more satisfaction
Joshi et al., 2024 (38)	TQLB vs. ESPB	26.4 ± 3.7 vs. 26.4 ± 3.8	56 vs.56	Elective C/S	II	SA	Total tramadol consumption in the first 48 h	[2, 3, 9, 10]	Clinical Trials Registry of India (CTRI/2022/02/040404)	The analgesic effect of bilateral ESPB at T12 was non-inferior to that of bilateral TQLB
Krohg et al., 2019 (39)	QLB vs. Sham block	34 ± 4 vs. 36 ± 4	20 vs.20	Elective C/S	I-III	SA	Ketobemidone consumption during the first 24 h postoperatively	[2, 4, 8]	ClinicalTrials.gov (identifier: NCT02036749)	QLB with ropivacaine reduces the postoperative ketobemidone consumption and pain intensity
Mostafa et al., 2023 (40)	TQLB vs. ESPB vs. No block	27 (23, 31) vs. 27 (22, 33) vs. 27 (23, 31)	51vs.50 vs.48	Elective C/S	I-II	SA	Time to first morphine requirement	[1, 2, 8]	ClinicalTrials.gov (NCT05254093)	Both QLB and ESPB provided superior analgesia and quality of recovery compared to the standard care, without significant difference between the two blocks
Priya et al., 2023 (43)	QLB-2 vs. ESPB	27.0 ± 4.0 vs. 28.0 ± 4.6	26vs.26	C/S	II	SA	Postoperative requirement for fentanyl in the first 24 h after surgery	[2, 8]	Clinical Trials Registry, India (CTRI/2021/09/036750)	QLB-II or ESPB reported similar analgesic efficacy, complications, and quality of recovery in the postoperative period
Qin et al., 2024 (3)	L-QLB-2 vs. Sham acupuncture	31.4 ± 3.9 vs. 31.1 ± 3.9	95vs.95	Elective C/S	I-II	CSEA	Pain scores on movement at 24 h	[2, 4, 8, 9]	Chinese Clinical Trial Registry (ChiCTR2300073559)	acupuncture did not lower postoperative pain scores or reduce analgesic medication consumption compared to L-QLB
Salama, 2020 (42)	QLB-2 vs. ITM vs. Sham block	31.1 ± 5.9 vs. 29.9 ± 7.5 vs. 32.5 ± 6.6	30vs.30 vs.30	Elective C/S	II	SA	IAS at rest and during movement	[1, 2, 3, 4, 8, 10]	PACTR201809600342881	QLB and ITM are effective analgesic regimens after CS. However, QLB provides better long-lasting analgesia and reduced total postoperative morphine consumption
Stopar-Pintaric et al., 2021 (4)	P-QLB vs. wound infiltration	31.9 ± 4.9 vs. 33.5 ± 4.7	58 vs.58	Elective C/S	II-III	SA	Opioid (piritramide) consumption at 24 h	[1, 2, 3, 4, 5, 8]	ClinicalTrials.gov identifier: NCT04000308	QLB was associated with lower 24-h opioid consumption compared with wound infiltration.
Verma et al., 2019 (43)	QLB vs. TAPB	30 ± 3 vs. 28 ± 3	30 vs.30	Elective C/S	I-II	SA	The time for rescue analgesic requirement	[1, 2]	Clinical Trials Registry- India (Registration No.: CTRI/2018/11/016420)	The QLB provided prolonged and effective analgesia in comparison to TAPB up to 72 h post-CS.
Yetik et al., 2022 (44)	QLB-2 vs. QLB-3	29.25 ± 5.41 vs. 29.63 ± 5.83	52vs.52	Elective C/S	II	GA	Pain scores evaluated at different time points postoperatively	[10]	ClinicalTrials.gov (Identifier: NCT04733313)	Although both QLBs were safe and reliable, QLB-3 provides more effective analgesia and patient satisfaction than QLB-2 in C/S
Yoshida et al., 2020 (45)	QLBi vs. Placebo	31 ± 6 vs. 30 ± 5	18vs.18	Elective C/S	II	SA	The elapsed time until the first postoperative analgesic use	[2, 9]	UMIN000036883	QLBi with the concentration and amount of local anesthetic used in the present study was clinically slightly effective, and the effect was limited for postoperative analgesia after CS

QLB, Quadratus lumborum block; Data are presented as mean ± standard deviation or median (Interquartile range 25,75). ESPB, Erector spinae plane block; C/S, Cesarean section; ASA, American Society of Anesthesiologists; UMIN, University Hospital Medical Information Network; GA, general anesthesia; TQLB, trans muscular quadratus lumborum block; TESPB, thoracic erector spinae plane block; SA, spinal anesthesia; LSCS, lower segment cesarean section; TFPB, transversalis facial plane block; TAPB, transversus abdominis plane blocks; ITM, intrathecal morphine; QLB-LSAL, quadratus lumborum block at the lateral supra-arcuate ligament; CSEA, combined spinal-epidural anesthesia; EA, epidural anesthesia; PCA, patient-controlled analgesia; L-QLB, lateral quadratus lumborum block; Secondary outcomes: [1] postoperative opioid consumption; [2] postoperative resting or movement pain scores; [3] time-to-first analgesic request; [4] first-ambulation time; [5] breastfeeding time; [6] PACU stay time; [7] hospital stay time; [8] opioid related complications (PONA, sedation, pruritus); [9] regional block related complications; [10] patients’ satisfaction; Secondary outcomes: [1] postoperative opioid consumption; [2] postoperative resting or movement pain scores; [3] time-to-first analgesic request; [4] first-ambulation time; [5] breastfeeding time; [6] PACU stay time; [7] hospital stay time; [8] opioid related complications (PONA, sedation, pruritus); [9] regional block related complications; [10] patients’ satisfaction.

**Table 2:** Details of block technique and analgesic regimens used in included studies.

Study	Groups	Pre-incisional analgesia	Supplemental postoperative analgesia	Block timing	Block technique	Local anesthetics
Ferit et al., 2021 (27)	QLB-2 vs. QLB-3	No premedication	PCA pump 1 g IV paracetamol at 8 hourly intervals Diclofenac sodium75 mg IM	After GA	USG bilaterally	0.3 mL. Kg-1 of 0.25% bupivacaine
Bilgin et al., 2023 (28)	anterior QLB vs. TFPB	No premedication	1 g of IV paracetamol 30 min before surgery and another 1 g every 8 h PCA pump	Before extubation at the end of surgery	USG bilaterally	25 mL of 0.25% bupivacaine
Blanco et al., 2015 (16)	QLB vs. Sham block	Oral ranitidine 150 mg	Rectal diclofenac 100mgand IV paracetamol 1 g PCA pump	At the end of surgery	USG bilaterally	1.0.125% bupivacaine 0.2 mlkg^−1^ 2. 0.9% normal saline 0.2 ml/kg^−1^
Blanco et al., 2018 (17)	QLB vs. TAPB	Oral ranitidine 150 mg	Rectal diclofenac (100 mg) and 1 g of IV paracetamol PCA pump	At the end of surgery	USG bilaterally	0.125% bupivacaine at 0.2 mL/kg in each side for a total of 0.4 mL/kg
Borys et al., 2021 (29)	QLB vs. TAPB	No premedication	Paracetamol, ketoprofen, diclofenac and tramadol Morphine (5 mg)	At the end of surgery	USG bilaterally	0.25% solution of bupivacaine 0.2 mL/kg
Borys et al., 2021 (30)	QLB vs. TAPB vs. No block	No premedication	PCA pump IV paracetamol	At the end of surgery	USG bilaterally	0.375% ropivacaine, 0.2 mL per kg (up to 20 mL), per side
Elashry et al., 2024 (31)	QLB-2 vs. QLB-3 vs. TAPB	No premedication	Paracetamol 15 mg/kg infusion/6 h A bolus of meperidine (0.5 mg/kg IV)	At the end of surgery	USG bilaterally	20 mL of bupivacaine 0.25% per side
Eldemrdash et al., 2025 (32)	QLB-2 vs. TFPB vs. TAPB	No premedication	-	-	USG bilaterally	20 mL of 0.25% bupivacaine per side
Giral et al., 2024 (33)	Posterior QLB vs. ITM	oral cimetidine 200 mg	IV paracetamol 1 g and ketoprofen 100 mg (h-1) PCA pump Oral Paracetamol 1 g and ketoprofen LP 100 mg Oral morphine	At the end of surgery	QLB: USG bilaterally	40 mL ropivacaine (20 mL/side) corresponding to a dose of 140 mg Morphine chlorhydrate 0.1 mg/mL was used in a unique intrathecal injection of 1 mL
Guo et al., 2022 (34)	QLB-LSAL vs. TQLB	No premedication	PCA pump	At the end of surgery	USG bilaterally	0.375% ropivacaine 20 mL per side
Hansen et al., 2018 (35)	TQLB vs. Sham block	No premedication	PCA pump 1 g paracetamol orally four times a day 400 mg ibuprofen orally three times a day	At the end of surgery	USG bilaterally	30 mL of ropivacaine 0.375% per side 30 mL saline 0.9% per side
Irwin et al., 2020 (36)	QLB vs. Sham block	No premedication	Rectal diclofenac 100 mg and IV Paracetamol 1 g PCA pump Oral paracetamol 1 g every 6 h and diclofenac 75 mg every 12 h Oxycodone 5 mg	At the end of surgery	USG bilaterally	20 mL levobupivacaine 0.25% per side Similar manner
Jadon et al., 2022 (37)	QLB vs. TAPB	metoclopramide (10 mg) and ranitidine (50 mg)	75 mg diclofenac IV 12-hourly Tramadol 50 mg IV	At the end of surgery	USG bilaterally	20 mL 0.375% ropivacaine per side
Joshi et al., 2024 (38)	TQLB vs. ESPB	oral pantoprazole 40 mg and metoclopramide 10 mg	Acetaminophen, starting with 1 g IV infusion at the conclusion of the block and every 8 h, and ketorolac 30 mg IV every 12 h for 2 days Tramadol 50 mg	At the end of surgery	USG bilaterally	20 mL of 0.25% ropivacaine per side
Krohg et al., 2019 (39)	QLB vs. Sham block	No premedication	Oral paracetamol 1 g and ibuprofen 400 mg, dosed together 4 times daily PCA pump	At the end of surgery	USG bilaterally	2 mg/mL ropivacaine per side Saline 9 mg/mL per side
Mostafa et al., 2023 (40)	TQLB vs. ESPB vs. No block	metoclopramide 10 mg, and ranitidine 50 mg	paracetamol (1 g/6 h) and diclofenac (50 mg/8 h) IV morphine	At the end of surgery	USG bilaterally	TQLB and ESPB received 20 mL of 0.25% bupivacaine per side
Priya et al., 2023 (43)	QLB-2 vs. ESPB	No premedication	Paracetamol 1 g IV every 6 h for 24 h Fentanyl 25 μg IV as rescue analgesia	At the end of surgery	USG bilaterally	0.25% bupivacaine 0.3 mL/kg per side
Qin et al., 2024 (3)	L-QLB-2 vs. Sham acupuncture	No premedication	1 g acetaminophen every 6 h and 75 mg diclofenac every 12 h PCA pump	At the end of surgery	L-QLB-2: USG bilaterally	20 mL of 0.33% ropivacaine per side
Salama, 2020 (42)	QLB-2 vs. ITM vs. Sham block	Oral ranitidine (150 mg)	IV paracetamol (1 g) and rectal diclofenac (100 mg) PCA pump	At the end of surgery	QLB-2: USG bilaterally	QLB-2 received 0.1 mL saline+ 24 mL 0.375% ropivacaine per side ITM received 0.1 mg morphine Sham block received 0.1 mL saline
Stopar-Pintaric et al., 2021 (4)	P-QLB vs. wound infiltration	No premedication	PCA pump	At the end of surgery	P-QLB: USG bilaterally	P-QLB received 20 mL 0.25% levobupivacaine per side+ 20 mL 0.9% saline into the surgical wound Wound infiltration received 20 mL of 0.25% levobupivacaine+P-QLB 20 mL 0.9% saline per side
Verma et al., 2019 (43)	QLB vs. TAPB	No premedication	IV tramadol in the bolus of 100 mg	At the end of surgery	USG bilaterally	0.2% ropivacaine 0.2 mL/kg
Yetik et al., 2022 (44)	QLB-2 vs. QLB-3	No premedication	PCA 1 g IV paracetamol at 8 hourly intervals Diclofenac sodium 75 mg IM	At the end of surgery	USG bilaterally	0.3 mL. Kg^−1^ 0.25% bupivacaine
Yoshida et al., 2020 (45)	QLBi vs. Placebo	-	1000 mg acetaminophen and 1 mg droperidol intravenously Flurbiprofen, acetaminophen, pentazocine and diclofenac	At the end of surgery	USG bilaterally	0.4 mL/kg of 0.25% ropivacaine Equal volume of 0.9% normal saline

QLB, Quadratus lumborum block; GA, General anesthesia; PCA, Patient controlled analgesia; IV, Intravenous; IM, Intramuscular; USG, Ultrasonography guided; TQLB, trans muscular quadratus lumborum block; TESPB, thoracic erector spinae plane block; SA, spinal anesthesia; LSCS, lower segment cesarean section; TFPB, transversalis facial plane block; TAPB, transversus abdominis plane blocks; ITM, intrathecal morphine; QLB-LSAL, quadratus lumborum block at the lateral supra-arcuate ligament; CSEA, combined spinal-epidural anesthesia; EA, epidural anesthesia; L-QLB, lateral quadratus lumborum block.

### 3.2 Quality assessment of the selected studies

As depicted in Supplementary Table 1, risk of bias was assessed using the Cochrane Risk of Bias 2 (RoB 2) tool across five domains: bias arising from the randomization process, bias due to deviations from intended interventions, bias due to missing outcome data, bias in measurement of the outcome, and bias in selection of the reported result. Overall judgments were classified as “low risk,” “some concerns,” or “high risk.” Detailed domain-level judgments for each study, including supporting quotations from the original publications regarding randomization, allocation concealment, blinding procedures, and outcome measurement methods, are provided in Supplementary Table 1. For studies judged as “some concerns” in the “bias in measurement of the outcome” domain (3, 31, 32, 42, 43), the primary concern was insufficient reporting on whether outcome assessors were blinded to group allocation during pain score assessment. For studies with sham blocks (Blanco 2015, Hansen 2018, Irwin 2020), we verified that sham procedures involved identical needle insertion and saline injection without local anesthetic, but the quality of participant blinding was not formally tested. In the “bias due to deviations from intended interventions” domain, all studies were rated low risk because no significant deviations from planned QLB techniques were reported. A total of 20 studies were characterized by an overall low risk of bias (4, 16, 17, 27–41, 44, 45), which signifies reliability in both methodology and outcomes. Three studies exhibit an unclear overall risk of bias (3, 42, 43), due to the no information on the measurement of the outcomes, which indicating that the reliability of their results cannot be adequately assessed based on the information provided. Notably, none of the studies demonstrate a high overall risk of bias.

Additionally, Supplementary Table 2 provides an overview of the certainty level associated with both primary and secondary outcomes. Assessed via the GRADE system, the quality of evidence for 24-h cumulative morphine consumption is considered high. For the remaining outcomes, the evidence quality is rated as moderate to high.

### 3.3 Primary outcomes

#### 3.3.1 Cumulative morphine consumption postoperatively (mg)

**Figure 2:**
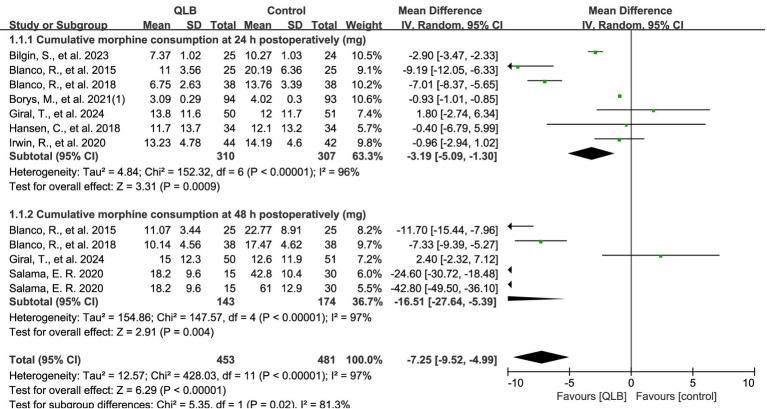
Forest plot of morphine consumption at 24 and 48 h postoperatively.

Seven studies investigated cumulative morphine consumption at 24 h postoperatively, revealing a statistically significant reduction in the QLB group compared with control [MD = −3.19; 95% CI: −5.09, −1.30; *p* = 0.0009; I^2^ = 96%]. However, the HKSJ-adjusted confidence interval was wider [−6.45, 0.07], crossing the null, reflecting uncertainty from high heterogeneity (16, 17, 28, 29, 33, 35, 36). Similarly, cumulative morphine consumption at 48 h postoperatively also favored in QLB compared with control group [MD = −16.51; 95%CI: −27.64, −5.39, *p* = 0.004, I^2^ = 97%] (16, 17, 33, 42) (Figure 2). Sensitivity analysis, conducted by sequentially removing one study (28), identified the source of heterogeneity, which remained low (Supplementary Table 3). Subgroup analysis revealed there is also no significant difference between QLB compared with sham block [MD = −3.73; 95%CI: −9.98, 2.51, *p* = 0.24, I^2^ = 91%] (16, 35, 36) (Supplementary Figure 1).

### 3.4 Secondary outcomes

#### 3.4.1 Postoperative pain scores at resting and movement

**Figure 3:**
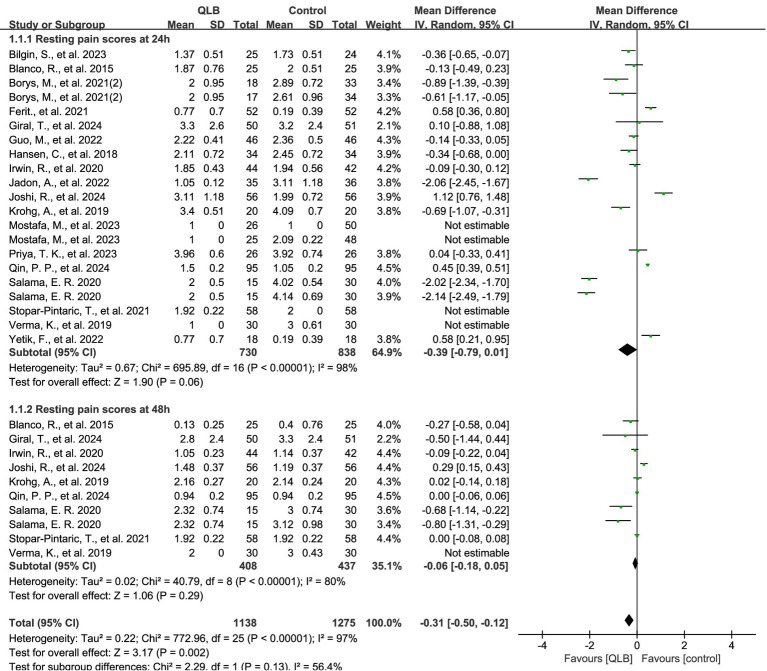
Forest plot of postoperative pain scores at resting at postoperatively.

No significant difference was observed in postoperative resting pain scores at 24 h (3, 4, 16, 27, 28, 30, 33–44) [MD = −0.39; 95% CI: −0.79, 0.01; *p* = 0.06; I^2^ = 98%] and 48 h (3, 4, 16, 33, 36, 38, 39, 42, 43) [MD = −0.06; 95% CI: −0.18, 0.05; *p* = 0.29; I^2^ = 80%] (Figure 3). Leave-one-out analysis identified Qin 2024 as an outlier; its removal rendered the 24 h resting pain difference significant (MD = −0.62; 95% CI: −1.12, −0.12), likely due to its sham acupuncture comparator.

**Figure 4:**
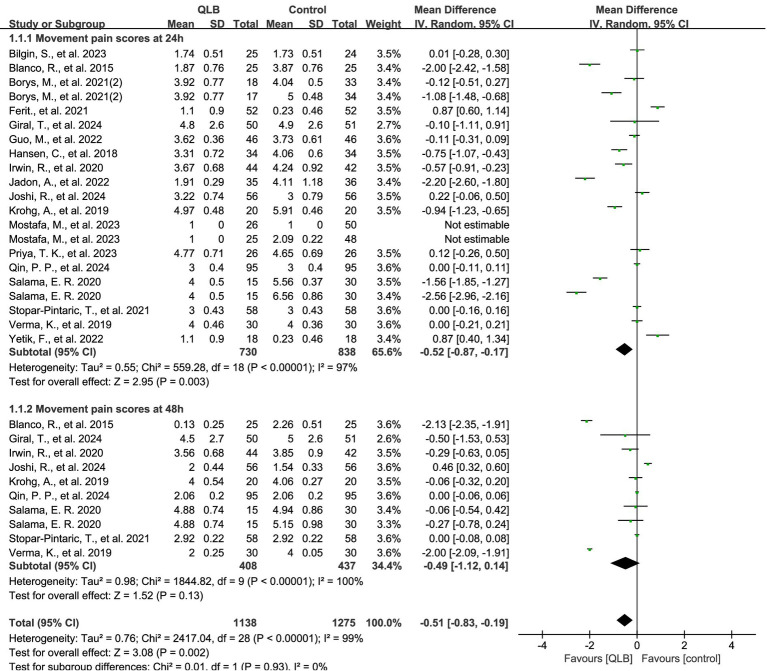
Forest plot of postoperative pain scores at movement at postoperatively.

Moreover, there is also no significant difference at the movement postoperative pain score at 48 h (3, 4, 16, 33, 36, 38, 39, 42, 43) [MD = −0.49; 95%CI: −1.12, 0.14, =0.13, I^2^ = 100%]. QLB showed a statistically significant reduction in postoperative movement pain at 24 h (3, 4, 16, 27, 28, 30, 33–44) [MD = −0.52; 95% CI: −0.87, −0.17; *p* = 0.003; I^2^ = 97%] (Figure 4). However, this 0.52-point reduction on a 0–10 scale is below the minimally clinically important difference (MCID) of 1.0–1.5 points, limiting its clinical relevance. The HKSJ-adjusted interval [−1.23, 0.19] crossed the null. The funnel plot revealed a slight asymmetric at resting and movement pain scores at 24 h and 48 h, respectively (Supplementary Figures 6, 7).

#### 3.4.2 Duration of surgical procedure and anesthesia

There is no significant difference observed in the duration of surgical procedure (3, 27, 31, 34, 36–38, 41, 43–45) [MD = 0.95; 95%CI: −0.40, 2.29, *p* = 0.17, I^2^ = 72%] and anesthesia time (3, 27, 32, 41, 44) [MD = −0.02; 95%CI: −3.98, 3.94, *p* = 0.99, I^2^ = 93%] (Supplementary Figure 2).

#### 3.4.3 Time to the first request post-operative analgesia and ambulation

There is no significant difference observed in the time to the first request post-operative analgesia (4, 27, 28, 31–35, 37, 38, 40, 42–45) [MD = 6.08; 95%CI: −0.65, 12.82, *p* = 0.08, I^2^ = 100%] and ambulation (3, 4, 35, 40, 42) [MD = 0.60; 95%CI: −0.14, 1.33, *p* = 0.11, I^2^ = 98%] (Supplementary Figure 3).

#### 3.4.4 Patient satisfaction score

Four studies investigated the patient satisfaction score between QLB and control group demonstrated there is no significant difference [MD = 0.15; 95%CI: −0.27, 0.56, *p* = 0.49, I^2^ = 98%] (32, 34, 37, 38) (Supplementary Figure 4).

#### 3.4.5 Opioid-related postoperative complications

There is no significant difference observed in PONV (31, 33, 34, 41) [MD = 0.89; 95%CI: 0.55, 1.45, *p* = 0.65, I^2^ = 0%] and postoperative hypotension (31, 33, 41) [MD = 1.30; 95%CI: 0.72, 2.40, *p* = 0.41, I^2^ = 0%] between QLB and control group (Supplementary Figure 5).

## 4 Discussion

This systematic review and meta-analysis of 23 RCTs involving 1,935 patients evaluates the analgesic efficacy of QLB compared with diverse regional techniques for postoperative pain after C/S (3, 4, 16, 17, 27–45). QLB was associated with statistically significant but modest reductions in 24-h and 48-h opioid consumption and 24-h dynamic pain. However, the effect sizes are small (MD = −3.19 mg morphine at 24 h; −0.52/10 pain points), the certainty of evidence is moderate-to-low due to very high heterogeneity (I^2^ > 90%), and HKSJ-adjusted confidence intervals cross the null for several outcomes. The clinical significance is limited, particularly given that the pain reduction is below the minimally clinically important difference (MCID) of 1.0–1.5 points. These findings should be interpreted cautiously, and QLB should be considered for selective use rather than routine application in all patients undergoing cesarean section.

The modest reductions in opioid consumption and movement pain observed with QLB may be partially explained by its anatomical positioning. As a truncal fascial plane block, QLB may contribute to analgesia through spread to the thoracic paravertebral space and lumbar plexus, potentially affecting both somatic and visceral pain pathways (16, 17, 46). However, the extent and consistency of paravertebral or sympathetic spread remain heterogeneous and somewhat controversial in the literature; some studies demonstrate limited cephalad spread, while others report extensive sensory blockade. The degree of visceral analgesia conferred by QLB is therefore uncertain, and the observed clinical effects may primarily reflect somatic blockade of the anterior abdominal wall, lower back, and pelvic region. This mechanistic uncertainty underscores the need for cautious interpretation of QLB’s purported advantages over somatic-targeting blocks such as TAPB (17, 31, 32, 37, 43).

Moreover, QLB could provide similar pain control, and quality of recovery compared with erector spinae plane block (ESPB) (38, 40, 41). Notably, subgroup analyses revealed no significant difference in 24-h morphine consumption between QLB and sham block, which may reflect the influence of co-administered multimodal analgesics (e.g., paracetamol, NSAIDs) or intrathecal morphine in some studies (16, 35, 36). However, the overall pooled effect consistently demonstrated QLB’s opioid-sparing benefit, supporting its role as an effective adjunct rather than a standalone analgesic. While the 24-h morphine reduction of 3.19 mg and dynamic pain reduction of 0.52 points are statistically significant, we acknowledge that their clinical significance is modest. The morphine reduction represents approximately 15–20% of typical 24-h consumption but may not translate to meaningful reductions in opioid-related adverse events. The pain score reduction is below the established MCID of 1.0–1.5 points for postoperative pain. Therefore, while QLB demonstrates a statistically detectable effect, the clinical relevance is uncertain, and QLB should be positioned as one component of a multimodal regimen rather than a standalone solution. This aligns with clinical practice, where QLB is typically integrated into a multimodal regimen to minimize opioid-related side effects such as nausea, vomiting, and sedation, though the current analysis did not detect significant differences in adverse events, possibly due to the low baseline incidence in the included studies (4). Moreover, our study excluded continuous QLB and QLB combined with adjuvant agents, interventions that have been shown to enhance and prolong the analgesic efficacy of QLB in previous research (47–51). This exclusionary criterion may have underestimated the full potential of QLB for post-cesarean pain management, as adjuvants (e.g., dexmedetomidine, clonidine) are known to extend the duration of local anesthetic action and reduce opioid requirements, while continuous QLB delivers sustained analgesia beyond the 48-h timeframe evaluated in our analysis. By focusing solely on single-shot QLB without adjuvants, our findings may not reflect the optimal clinical application of this block technique, highlighting a key consideration for interpreting the generalizability of our results.

Direct comparisons between QLB and other regional blocks highlight important clinical trade-offs, though these comparisons are limited by high heterogeneity and comparator-dependent effects. In subgroup analyses, QLB showed larger opioid-sparing effects when compared with TAPB and wound infiltration, but non-significant differences when compared with ESPB and intrathecal morphine. The attenuation of QLB’s effect in the presence of intrathecal morphine (*p*-interaction = 0.04) suggests that QLB may offer limited additive benefit when neuraxial opioids are already administered. For patients receiving spinal anesthesia without intrathecal opioids, or those with contraindications to neuraxial opioids, QLB may provide modest additional analgesia. However, given the modest effect sizes and moderate-to-low certainty evidence, QLB should not be regarded as universally superior to alternative regional techniques (17, 31–33, 37, 38, 40, 41, 43).

The findings of this meta-analysis may inform post-C/S analgesia within ERAS frameworks, though the implications should be interpreted cautiously. The modest reductions in opioid consumption and dynamic pain may indirectly support recovery goals such as early ambulation and breastfeeding initiation; however, these endpoints did not reach statistical significance in the current analysis, likely due to insufficient statistical power and the dominant influence of other ERAS components (e.g., early mobilization protocols, nutrition support). The very high residual heterogeneity across subgroups (I^2^ > 85% in most analyses), significant comparator-dependent effects, and attenuation of efficacy with intrathecal morphine co-administration indicate that the pooled estimate represents an average effect across highly heterogeneous clinical contexts rather than a universally applicable effect. For anesthesiologists, QLB may be selectively considered as part of multimodal analgesia for patients receiving spinal anesthesia without intrathecal opioids, or those with opioid intolerance. However, given the moderate-to-low GRADE certainty and modest clinical effect sizes, routine mandatory use of QLB in all patients undergoing cesarean section is not supported by current evidence. The ultrasound-guided technique is standardized across most included studies, but proper training in anatomical localization and injection technique remains essential.

### 4.1 GRADE certainty and implications for practice

The GRADE certainty for the primary outcome (24-h morphine consumption) is moderate (downgraded for serious inconsistency, I^2^ = 96%), and low for 48-h morphine and pain scores (downgraded for very serious inconsistency and imprecision). This indicates limited confidence in the true effect estimates, and future studies may substantially change these estimates. The pooled effect should be interpreted as an average across highly heterogeneous clinical contexts rather than a universally applicable effect. For clinical practice, QLB may be selectively considered as part of multimodal analgesia for patients without intrathecal morphine co-administration or those with opioid intolerance. However, given the modest effect sizes, high residual heterogeneity, and comparator-dependent efficacy, routine mandatory use of QLB in all patients undergoing cesarean section is not supported by current evidence.

### 4.2 Clinical significance versus statistical significance

While the 24-h morphine reduction of 3.19 mg and dynamic pain reduction of 0.52 points are statistically significant, their clinical significance is modest and uncertain. The morphine reduction represents approximately 15–20% of typical 24-h consumption, but this 3 mg difference may not translate into clinically meaningful reductions in opioid-related adverse events such as nausea, vomiting, or sedation, particularly given that no significant differences in PONV or hypotension were observed. The dynamic pain reduction of 0.52 points on a 0–10 scale falls well below the commonly accepted MCID threshold of 1.0–1.5 points for postoperative pain, suggesting that patients may not perceive this difference as clinically meaningful. The distinction between statistical significance and patient-relevant benefit should remain central to clinical decision-making: QLB demonstrates a detectable but modest effect that may not improve patient-centered outcomes such as satisfaction, quality of recovery, or maternal–infant bonding.

### 4.3 Limitations

Despite its strengths, this meta-analysis has several limitations that should be considered when interpreting the results. First, very high residual heterogeneity (I^2^ > 85% even within subgroups) remains the most critical limitation. Despite comprehensive subgroup analyses by QLB type, comparator type, intrathecal morphine use, and anesthesia type, no single subgroup fully resolved heterogeneity. This indicates that the pooled effect estimate represents an average across highly heterogeneous clinical contexts—including variations in QLB techniques, local anesthetic doses, multimodal analgesic regimens, comparator types, and patient populations—rather than a universally applicable effect. The significant effect modification by comparator type (*p*-interaction < 0.001) and intrathecal morphine co-administration (*p*-interaction = 0.04) further underscores that QLB’s efficacy is context-dependent and cannot be generalized across all clinical scenarios. Second, five studies raised some concerns in RoB 2 assessments, primarily due to insufficient reporting of outcome assessor blinding. While no studies were rated as high risk, this uncertainty may introduce bias in pooled estimates. We have downgraded GRADE certainty accordingly. Third, the analysis focused on short-term outcomes (≤48 h). Data on chronic postsurgical pain (CPSP), postpartum depression, quality of recovery (QoR-15), maternal–infant bonding, and breastfeeding duration were scarce or absent. Only 2 studies reported outcomes beyond 3 months, with inconsistent results. Given that C/S is a known risk factor for CPSP (incidence 6–18% at 12 months) and that acute pain severity influences postpartum depression risk, future studies must evaluate these patient-centered long-term outcomes. The absence of validated PROMs is a critical gap in the current evidence base. Fourth, we did not search grey literature or non-English publications, and 70 full-text articles were inaccessible. While sensitivity analyses suggested minimal impact, this may have introduced selection bias. Fifth, all QLB procedures were post-incisional, precluding evaluation of pre-emptive versus post-incisional timing. Sixth, the modest clinical significance of statistically significant findings (below MCID for pain, uncertain clinical impact of 3 mg morphine reduction) suggests that QLB’s value lies in multimodal integration rather than standalone efficacy.

## 5 Conclusion

In summary, this systematic review and meta-analysis demonstrates that QLB was associated with statistically significant but modest reductions in postoperative opioid consumption (24 h: MD = −3.19 mg; 48 h: MD = −16.51 mg) and 24-h dynamic pain (MD = −0.52/10) after cesarean section. However, the clinical significance is limited: the pain reduction falls below the minimally clinically important difference (1.0–1.5 points), and the 3 mg morphine reduction may not translate into meaningful reductions in opioid-related adverse events. The certainty of evidence is moderate for 24-h morphine consumption and low for 48-h morphine and pain scores, due to very high heterogeneity (I^2^ > 90%) and wide confidence intervals that cross the null for several outcomes.

QLB’s modest opioid-sparing effect aligns with ERAS principles; however, the effect is comparator-dependent and attenuated by intrathecal morphine co-administration. QLB-3 (transmuscular) may offer greater efficacy than QLB-2 (posterior), though residual heterogeneity remains high even within subgroups. The pooled estimate should be interpreted as an average effect across highly heterogeneous clinical contexts rather than a universally applicable effect. QLB should be considered as a selective adjunct in multimodal analgesia, particularly for patients without intrathecal morphine or those with opioid intolerance, rather than a routine mandatory intervention for all patients undergoing cesarean section.

*Future research priorities include*: (1) standardized QLB techniques and dosing protocols to reduce heterogeneity; (2) evaluation of long-term outcomes (CPSP, postpartum depression, maternal–infant bonding) and patient-centered outcomes using validated PROMs; (3) systematic safety monitoring for local anaesthetic systemic toxicity; (4) comparison of pre-emptive versus post-incisional timing; (5) head-to-head RCTs under standardized multimodal regimens to enable definitive comparisons and network meta-analysis; and (6) studies specifically designed to detect clinically meaningful differences in opioid-related adverse events. For clinical practice, QLB may be selectively considered for patients receiving spinal anesthesia without intrathecal opioids or those with opioid intolerance, but routine mandatory use in all patients undergoing cesarean section is not supported by current moderate-to-low certainty evidence.

## Data Availability

The original contributions presented in the study are included in the article/Supplementary material, further inquiries can be directed to the corresponding author.
