# Supplementary material for: Comparison of ultrasound-guided quadratus lumborum block and other regional blocks for postoperative pain in cesarean section: a systematic review and meta-analysis of randomized clinical trials
Source: Front Med (Lausanne). 2026 Jul 9;13:1861119. doi: 10.3389/fmed.2026.1861119 (PMC13392928; doi:10.3389/fmed.2026.1861119)
Supplement: Supplementary file 1 [file Supplementary_file_1.doc]

### Pubmed,2025-09-20,57

| #1:**((((((((((("Cesarean Section"[Mesh]) OR (Cesarean Sections[Title/Abstract])) OR (Abdominal Delivery[Title/Abstract])) OR (C-Section (OB[Title/Abstract]))) OR (C Section (OB[Title/Abstract]))) OR (C-Sections (OB[Title/Abstract]))) OR (Caesarean Section[Title/Abstract])) OR (Caesarean Sections[Title/Abstract])) OR (Delivery, Abdominal[Title/Abstract])) OR (Abdominal Deliveries[Title/Abstract])) OR (Deliveries, Abdominal[Title/Abstract])) OR (Postcesarean Section[Title/Abstract])** |
| --- |
| #2:**Quadratus lumborum block[Title/Abstract]** |
| #3:#1 AND #2 |
