# Supplementary material for: Comparison of ultrasound-guided quadratus lumborum block and other regional blocks for postoperative pain in cesarean section: a systematic review and meta-analysis of randomized clinical trials
Source: Front Med (Lausanne). 2026 Jul 9;13:1861119. doi: 10.3389/fmed.2026.1861119 (PMC13392928; doi:10.3389/fmed.2026.1861119)
Supplement: Supplementary file 2 [file Supplementary_file_2.doc]

Supplementary fig.1. Subgroup analysis of forest plot of cumulative morphine consumption at 24 h between QLB and sham block.

Supplementary fig.2. Forest plot of duration of surgical procedure and anesthesia.

Supplementary fig.3. Forest plot of time to the first request post-operative analgesia and ambulation.

Supplementary fig.4. Forest plot of patient satisfaction score.

Supplementary fig.5. Forest plot of opioid-related postoperative complications.

Supplementary fig.6. Funnel plot of resting pain scores at 24 and 48 h postoperatively.

Supplementary fig.7. Funnel plot of movement pain scores at 24 and 48 h postoperatively.
