# Supplementary material for: Comparison of ultrasound-guided quadratus lumborum block and other regional blocks for postoperative pain in cesarean section: a systematic review and meta-analysis of randomized clinical trials
Source: Front Med (Lausanne). 2026 Jul 9;13:1861119. doi: 10.3389/fmed.2026.1861119 (PMC13392928; doi:10.3389/fmed.2026.1861119)
Supplement: Supplementary file 3 [file Table_1.doc]

Supplementary Table 1. Risk bias of assessment of included studies.

| Study | Bias arising from the randomization process | Bias due to deviations from intended interventions | Bias due to missing outcome data | Bias in measurement of the outcome | Bias in selecting the reported result | Overall bias |
| --- | --- | --- | --- | --- | --- | --- |
| Ferit., et al. 2021 | Low | Low | Low | Low | Low | Low risk of bias |
| Bilgin, S., et al. 2023 | Low | Low | Low | Low | Low | Low risk of bias |
| Blanco, R., et al. 2015 | Low | Low | Low | Low | Low | Low risk of bias |
| Blanco, R., et al. 2018 | Low | Low | Low | Low | Low | Low risk of bias |
| Borys, M., et al. 2021(1) | Low | Low | Low | Low | Low | Low risk of bias |
| Borys, M., et al. 2021(2) | Low | Low | Low | Low | Low | Low risk of bias |
| Elashry, H. E., et al. 2024 | Low | Low | Low | Low | Low | Low risk of bias |
| Eldemrdash, A. M., et al. 2025 | Low | Low | Low | Low | Low | Low risk of bias |
| Giral, T., et al. 2024 | Low | Low | Low | Low | Low | Low risk of bias |
| Guo, M., et al. 2022 | Low | Low | Low | Low | Low | Low risk of bias |
| Hansen, C., et al. 2018 | Low | Low | Low | Low | Low | Low risk of bias |
| Irwin, R., et al. 2020 | Low | Low | Low | Low | Low | Low risk of bias |
| Jadon, A., et al. 2022 | Low | Low | Low | Low | Low | Low risk of bias |
| Joshi, R., et al. 2024 | Low | Low | Low | Low | Low | Low risk of bias |
| Krohg, A., et al. 2019 | Low | Low | Low | Low | Low | Low risk of bias |
| Mostafa, M., et al. 2023 | Low | Low | Low | Low | Low | Low risk of bias |
| Priya, T. K., et al. 2023 | Low | Low | Low | Low | Low | Low risk of bias |
| Qin, P. P., et al. 2024 | Low | Low | Low | Unclear | Low | Unclear risk of bias |
| Salama, E. R. 2020 | Low | Low | Low | Unclear | Low | Unclear risk of bias |
| Stopar-Pintaric, T., et al. 2021 | Low | Low | Low | Low | Low | Low risk of bias |
| Verma, K., et al. 2019 | Low | Low | Low | Unclear | Low | Unclear risk of bias |
| Yetik, F., et al. 2022 | Low | Low | Low | Low | Low | Low risk of bias |
| Yoshida, K., et al. 2020 | Low | Low | Low | Low | Low | Low risk of bias |

Note: **Domain-level justifications:**

• **Bias arising from the randomization process** (Low risk, all 23 studies): All studies reported random sequence generation (computer-generated randomization or random number tables) and allocation concealment (sealed opaque envelopes or centralized pharmacy randomization). No study showed baseline imbalances suggesting inadequate randomization.

• **Bias due to deviations from intended interventions** (Low risk, all 23 studies): All studies reported that participants received the intended QLB or comparator intervention as randomized. No significant protocol deviations were reported. However, we note that blinding of participants to sham procedures was not formally verified in any study (e.g., through post-intervention questioning), which represents a potential but unquantified risk of performance bias.

• **Bias due to missing outcome data** (Low risk, all 23 studies): All studies reported complete outcome data or used intention-to-treat analysis with minimal missing data (<5% per study).

• **Bias in measurement of the outcome** (Low risk: 18 studies; Some concerns: 5 studies): The 18 low-risk studies explicitly reported that outcome assessors were blinded to group allocation during pain score assessment. The 5 studies with some concerns (Qin 2024, Salama 2020, Verma 2019, Elashry 2024, Eldemrdash 2025) did not provide sufficient detail on outcome assessor blinding, raising the possibility of detection bias in pain score recording.

• **Bias in selection of the reported result** (Low risk, all 23 studies): All studies reported pre-specified outcomes as described in their protocols or methods sections, with no evidence of selective reporting.

Supplementary Table 2. Quality of evidence assessment for main results.

| Outcomes | QLB *vs.* control | Corresponding risk of QLB in different outcomes  *vs.* control | Certainty |
| --- | --- | --- | --- |
| Morphine consumption at 24 postoperatively | 310 *vs.* 307 | -3.19 (-5.09, -1.30) | ⨁⨁⨁⨁ High |
| Morphine consumption at 48 postoperatively | 143 *vs.* 174 | -16.51 (-27.64, -5.39) | ⨁⨁⨁⨁ High |
| Postoperative pain scores at resting at 24h | 730 *vs.*838 | -0.39 (-0.79, -0.01) | ⨁⨁⨁◯ Moderate |
| Postoperative pain scores at resting at 48h | 408 *vs.*437 | -0.06 (-0.18, 0.05) | ⨁⨁⨁◯ Moderate |
| Postoperative pain scores at movement at 24h | 730 *vs.*838 | -0.52 (-0.87, -0.17) | ⨁⨁⨁◯ Moderate |
| Postoperative pain scores at movement at 48h | 408 *vs.*437 | -0.49 (-1.12, 0.14) | ⨁⨁⨁◯ Moderate |
| Duration of surgical procedure | 474 *vs.*493 | 0.95 (-0.40, 2.29) | ⨁⨁⨁⨁ High |
| Duration of anesthesia | 261 *vs.*297 | -0.02 (-3.98, 3.94) | ⨁⨁⨁⨁ High |
| Time to the first request post-operative analgesia | 593 *vs.*727 | 6.08 (-0.65, 12.82) | ⨁⨁⨁⨁ High |
| Time to the ambulation | 268 *vs.*345 | 0.60 (-0.14, 1.33) | ⨁⨁⨁⨁ High |
| Patient satisfaction score | 173 *vs.*210 | 0.15 (-0.27, 0.56) | ⨁⨁⨁⨁ High |
| PONV | 142 *vs.*163 | 1.30 (0.70, 2.40) | ⨁⨁⨁⨁ High |
| Hypotension | 96 *vs.*117 | 0.89 (0.55, 1.45) | ⨁⨁⨁⨁ High |

Supplementary Table 3. Sensitivity analysis of postoperative cumulative morphine consumption at 24h between QLB and control group.

| Study | Z effect | MD | 95%CI | I^2^ | P value |
| --- | --- | --- | --- | --- | --- |
| Bilgin, S., et al. 2023 | 1.85 | -3.07 | -6.31, 0.18 | 95% | 0.06 |
| Blanco, R., et al. 2015 | 2.44 | -2.32 | -4.19, -0.46 | 96% | 0.01^*^ |
| Blanco, R., et al. 2018 | 2.81 | -2.39 | -4.06, -0.72 | 94% | 0.005^*^ |
| Borys, M., et al. 2021(1) | 2.69 | -3.59 | -6.20, -0.98 | 91% | 0.007^*^ |
| Giral, T., et al. 2024 | 3.64 | -3.70 | -5.69, -1.70 | 97% | 0.0003^*^ |
| Hansen, C., et al. 2018 | 3.37 | -3.37 | -5.33, -1.41 | 97% | 0.0008^*^ |
| Irwin, R., et al. 2020 | 3.33 | -3.61 | -5.71, -1.48 | 97% | 0.0009^*^ |
